# Editorial: Emerging concepts for respiratory viruses after the pandemic

**DOI:** 10.3389/fcimb.2025.1758434

**Published:** 2025-12-16

**Authors:** Dimitra Dimopoulou, Chrysanthi Skevaki, Fusun Can, Florence Morfin-Sherpa, Paraskevi C. Fragkou

**Affiliations:** 1 Second Department of Pediatrics, “P. & A. Kyriakou” Children’s Hospital, National and Kapodistrian University of Athens, School of Medicine, Athens, Greece; 2 European Society of Clinical Microbiology and Infectious Diseases (ESCMID) Study Group for Respiratory Viruses (ESGREV), Basel, Switzerland; 3 Department of Environmental Health, Harvard T.H. Chan School of Public Health, Boston, MA, United States; 4 Institute of Laboratory Medicine and Pathobiochemistry, Molecular Diagnostics, University of Marburg, Marburg, Germany; 5 German Centre for Lung Research - Deutsches Zentrum für Lungenforschung (DZL), The Universities of Giessen and Marburg Lung Center (UGMLC), Giessen, Germany; 6 Centre for Public Health Research and Education (CPHRE), Academy of Athens, Athens, Greece; 7 Department of Medical Microbiology, School of Medicine, Koç University, Istanbul, Türkiye; 8 Koç University İşBank Center for Infectious Diseases, Koç University Hospital (KUISCID), Istanbul, Türkiye; 9 Laboratory of Virology, Institut des Agents Infectieux, Hospices Civils de Lyon, Lyon, France; 10 CIRI (Centre International de Recherche en Infectiologie), Team VirPatH, Univ Lyon, Institut national de la santé et de la recherche médicale (INSERM) U1111, Centre national de la recherche scientifique (CNRS) UMR5308, Université Claude Bernard Lyon 1, École Normale Supérieure (ENS) de Lyon, Lyon, France; 11 Internal Medicine Department, Aegli Medical Clinic, Athens, Greece

**Keywords:** artificial intelligence - AI, diagnostics, epidemiology, long-term sequelae, pathogenesis, research tools and models, respiratory viruses

Respiratory virus research has been reshaped by the COVID-19 pandemic and the effects of climate change, both of which contributed to a shift in the epidemiology of respiratory viruses and highlighted gaps in our understanding about the plasticity and long-term consequences of these changes (Chow et al., 2023; He et al., 2023). The pandemic also accelerated advances in research models and methods, underscoring evolving seasonality patterns, viral pathogenesis, and host interactions (Chow et al., 2023; He et al., 2023). This Research Topic explored the changing epidemiology and pathogenesis of respiratory viruses, focusing on seasonality, long-term outcomes, and host responses. It also emphasized advances in diagnostics and therapeutics and the use of artificial intelligence for predictive modeling, aiming to inform public health strategies in the post-pandemic era.

## Epidemiology and seasonality of respiratory infections in the post-COVID-19 era

The COVID-19 pandemic has significantly impacted the epidemiology of respiratory viruses due to widespread public health interventions such as lockdowns, masking, social distancing and school closures that resulted in the interruption of the SARS-CoV-2 transmission and the seasonal dynamics of other respiratory viruses (Chen and Er, 2022; Principi et al., 2023). Alzahrani et al. reported the prominence of rhinovirus/enterovirus, SARS-CoV-2, respiratory syncytial virus (RSV), and adenovirus in respiratory infections in Saudi Arabia, with clear winter peaks and age-specific susceptibility, highlighting the significant burden of respiratory infections, particularly among pediatric populations, the impact of public health interventions, such as COVID-19 restrictions, on the transmission of respiratory viruses and the need for targeted interventions. Similarly, in Mexico, De Arcos-Jimenez et al. found that influenza experienced a sharp decline during the pandemic but rebounded post-pandemic, RSV demonstrated a delayed resurgence, while other respiratory viruses exhibited heterogeneous rebound patterns. Importantly, advanced age, male sex, cardiovascular disease, obesity, and immunosuppression were major risk factors for severe acute respiratory infection (SARI), with vaccination offering consistent protection, reinforcing its crucial role in respiratory infection mitigation strategies. Thus, the dynamic interplay between viral competition, public health interventions, and human behavior likely contributed to the observed changes in SARI trends, emphasizing the need for ongoing surveillance and adaptive public health responses. Data from China further reinforce this pattern; Yue et al. showed that non-pharmaceutical interventions against COVID-19 markedly restricted the spread of influenza, adenovirus, and Mycoplasma pneumoniae in children, while also reduced bacterial co-infections. Following the end of China’s “Zero-COVID” policy, atypical seasonal patterns and rising co-infections were observed, with respiratory viruses resuming circulation. These findings reflect the dual impact of population immunity gaps and viral re-emergence. Furthermore, Li et al. highlighted that in China, SARS-CoV-2 and influenza dominated respiratory infections in adults, while RSV remained predominant in children and influenza in school-aged children, underscoring shifting age distributions. These findings underscore the need for continuous multi-pathogen surveillance and vaccination strategies to prevent future epidemic surges.

In addition to the shifts in viral seasonality, important pathogen-specific and clinical insights that inform pediatric health policy are also highlighted. Lu et al. provide evidence that despite a global 76.8% reduction in childhood mortality due to lower respiratory tract infection (LRTI) since 1990, LRTIs remain a leading cause of death in children from low- and middle-income countries, with Streptococcus pneumoniae, Staphylococcus aureus, and *Klebsiella pneumoniae* remaining dominant pathogens, while among newborns, the leading pathogens were Klebsiella pneumoniae, Group B Streptococcus, and *Acinetobacter baumannii*. This study further predicts rising influenza-related mortality by 2031, reinforcing the urgency of expanding vaccine coverage, improving sanitary conditions and early interventions for high-risk children in order to reduce LRTI burden. Fang et al. provides molecular insights, demonstrating that specific adenovirus (HAdV) genotypes, such as HAdV-3, HAdV-7, HAdV-2, and HAdV-1 were the predominant HAdV types in children hospitalized with respiratory infections in China, and particularly HAdV-7 is linked to severe pediatric pneumonia, highlighting the role of viral genetic variability in determining disease severity and clinical outcome and the need to differentiate HAdV types for both epidemiological surveillance and clinical management. Finally, Fu et al. report high reinfection rates of nearly 30% within five months among primary healthcare workers in China during the Omicron wave, particularly among women, nurses, those with fever clinic experience and working over 8 hours per day, underscoring occupational risks and the need for targeted protective measures, including appropriate work arrangements, regular health monitoring, and the promotion of healthy lifestyle habits.

To conclude, these studies demonstrate that the pandemic reshaped the epidemiology, seasonality, and clinical impact of respiratory viruses, and they highlight the ongoing vulnerabilities across pediatric and healthcare populations, as well as the need for continuous enhanced surveillance, vaccination, and targeted protective strategies to address future respiratory viral infection threats in the post-pandemic era.

## Immunology, pathogenesis, and therapeutic advances in COVID-19

While a huge body of data on COVID-19 pathogenesis is currently available, many aspects of the complex host-pathogen interplay remain still unknown. Bugatti et al. showed that SARS-CoV-2 can infect ACE2-negative primary human lung microvascular endothelial cells (HL-mECs) through an alternative entry mechanism that relies on the Arg-Gly-Asp (RGD) motif expressed in the receptor binding domain (RBD) of the spike protein in the viral spike protein. Viral entry into endothelial cells triggers phenotypic remodeling and angiogenic responses, contributing to vascular dysfunction observed in COVID-19. As RGD motif is not naturally exposed on the spike protein, heparan sulfate proteoglycans (HSPGs) function as essential cofactors that expose the RGD site, enabling spike interaction with αvβ3 integrin, thus HSPGs are central to SARS-CoV-2 infection of endothelial cells and its associated dysfunction, signifying them as a potential therapeutic target to mitigate endothelial involvement in COVID-19.

Understanding the pathogenetic mechanisms underlying respiratory viral infections requires the use of advanced single-cell technologies. Mass cytometry or cytometry by time-of-flight (CyTOF) offers this possibility by enabling simultaneous measurement of more than 50 markers per cell, allowing comprehensive phenotypic and functional profiling of immune populations (Zunder et al., 2022). In this setting, Balz et al. introduced a novel CyTOF protocol optimized for the immunophenotyping of low-frequency antigen-specific T cells. Using this enhanced workflow, they assessed virus-specific T-cell responses in SARS-CoV-2–vaccinated, SARS-CoV-2–infected, and non-exposed healthy individuals through high-parameter mass cytometry. Their analysis revealed a marked phenotypic heterogeneity among antigen-responsive CD4^+^, CD8^+^, and γδ T-cell subsets following antigen-specific stimulation, highlighting the protocol’s ability to resolve diverse functional T-cell states even within rare populations. Overall, this paper establishes a dual-barcoding, debris-free CyTOF protocol for reliable detection of extremely rare antigen-specific T cells; this protocol allows robust, high-content immunophenotyping of scarce T-cell responses in both mouse and human samples.

Regarding the treatment of COVID-19, the effectiveness of several therapeutic interventions patients has been evaluated since the onset of the pandemic. While evidence-based guidelines are now in place for both community dwellers and hospitalized patients, research in potential new or repurposed molecules remains ongoing. Xu et al., found in their retrospective study among 264 hospitalized COVID-19 patients, that azvudine significantly reduced both composite disease progression and all-cause mortality compared to standard care alone. Patients receiving azvudine also showed better improvement in oxygen saturation during hospitalization. These results highlight a potential therapeutic benefit of azvudine among COVID-19 hospitalized patients which should be further investigated and validated in randomized controlled trials.

Besides treatment, identifying predictive tools which stratify patients according to their risk for increased disease severity and adverse outcomes is crucial for the optimal management of COVID-19 patients. Tang et al. conducted a retrospective study among 137 Intensive Care Unit (ICU) patients with critical COVID-19 during the Omicron period; they found that lactate levels and the weekly lymphocyte change rate are strong predictors of mortality in ICU patients with severe COVID-19. A lactate threshold of 1.75 mmol/L effectively identified high-risk patients with significantly poorer survival. Combining lactate with lymphocyte dynamics improved prognostic accuracy. The neutrophil-to-lymphocyte ratio partially mediated lactate’s effect on mortality, indicating an interaction between inflammation and metabolic stress. Respiratory parameters did not predict outcomes, suggesting that elevated lactate reflects microcirculatory dysfunction rather than ventilation failure. Although these biomarkers should be further evaluated in prospective studies, it may represent a useful tool for critical COVID-19.

Vaccination against respiratory viruses (when available) remains the cornerstone of prevention, particularly among high-risk populations. However, data on long-term immunity in immunocompromised children who received SARS-CoV-2 vaccines remain limited. Russo et al. conducted a prospective observational study that demonstrated Vaccinated children maintained immune memory up to 17 months after receiving COVID-19 vaccines, but immunocompromised children showed significantly weaker immunity than healthy children. Immunocompromised children had lower anti-spike IgG levels and markedly reduced neutralizing antibodies, especially against Omicron BA.5 and after BBIBP-CorV vaccination. While CD4^+^ T-cell memory was similar between groups, CD8^+^ T-cell responses and IFN-γ production were notably diminished in immunocompromised children. These findings denote the need for updated vaccines and improved booster coverage.

From a public health perspective, important insights were gained during the COVID-19 pandemic regarding optimal policies to control viral transmission. Policymakers and stakeholders implemented measures often without solid evidence of their effectiveness. In this context, Artificial Intelligence (AI) proves extremely valuable, as it can generate predictive models to evaluate the impact of public health interventions. To this end, Dong et al. developed a detailed agent-based COVID-19 simulation model for a Chinese town (Xi’an) using real geographic, census, and mobility data. The model accurately reproduced the city’s 2021–2022 outbreak, with simulated case numbers closely matching official reports. By integrating individual behaviors and government measures such as testing, lockdowns, and mask use, it assessed how interventions affected disease spread. Scenario analyses, including a 100% mask-wearing simulation, showed modest reductions in cases. Overall, the model provides a powerful tool for predicting epidemic trends and supporting public health decision-making.

## Conclusions

Taken together, the studies featured in this Research Topic offer a broad, yet detailed picture of how the pandemic has reshaped our understanding of respiratory viruses—from shifting in their circulation patterns to the emergence of new clinical and immunological insights. As we move forward, these observations emphasize the importance of flexible surveillance systems, thoughtful public health planning, and continued scientific investment to anticipate and respond to future challenges.
